# NDs@PDA@ICG Conjugates for Photothermal Therapy of Glioblastoma Multiforme

**DOI:** 10.3390/biomimetics4010003

**Published:** 2019-01-11

**Authors:** Damian Maziukiewicz, Bartosz F. Grześkowiak, Emerson Coy, Stefan Jurga, Radosław Mrówczyński

**Affiliations:** 1 NanoBioMedical Centre, Adam Mickiewicz University, ul. Umultowska 85, PL-61614 Poznań, Poland; damian.maziukiewicz@amu.edu.pl (D.M.); bartoszg@amu.edu.pl (B.F.G.); coyeme@amu.edu.pl (E.C.); stjurga@amu.edu.pl (S.J.); 2 Department of Macromolecular Physics, Faculty of Physics, Adam Mickiewicz University, Umultowska 85, PL-61614 Poznań, Poland

**Keywords:** nanodiamonds, polydopamine, glioblastoma multiforme, indocyanine green, photothermal therapy, cancer treatment

## Abstract

The growing incidence of cancer is a problem for modern medicine, since the therapeutic efficacy of applied modalities is still not satisfactory in terms of patients’ survival rates, especially in the case of patients with brain tumors. The destructive influence of chemotherapy and radiotherapy on healthy cells reduces the chances of full recovery. With the development of nanotechnology, new ideas on cancer therapy, including brain tumors, have emerged. Photothermal therapy (PTT) is one of these. It utilizes nanoparticles (NPs) that can convert the light, preferably in the near-infrared (NIR) region, into heat. In this paper, we report the use of nanodiamonds (NDs) conjugated with biomimetic polydopamine (PDA) and indocyanine green (ICG) for glioblastoma cancer PTT therapy. The obtained materials were thoroughly analyzed in terms of their PTT effectiveness, as well as their physicochemical properties. The performed research demonstrated that NDs@PDA@ICG can be successfully applied in the photothermal therapy of glioblastoma for PTT and exhibited high photothermal conversion efficiency *η* above 40%, which is almost 10 times higher than in case of bare NDs. In regard to our results, our material was found to lead to a better therapeutic outcome and higher eradication of glioblastoma cells, as demonstrated in vitro.

## 1. Introduction

Constant development of nanotechnology allows for the production of materials which can be utilized in many areas of science. The engineering of those span from electronics to medicine [1], where the biomedical aspects of nanomaterials are of particular interest [2,3]. Properly designed nanomaterials can serve as scaffolds [4,5], drug [6] or gene [7] carriers, or due to their inherent properties, can be utilized in anticancer therapy [8,9,10]. With constantly growing numbers of cancer incidence [11], tumor treatment has become one of the most investigated topics all around the world. With current technology, we are limited to therapies that include surgical resection, chemotherapy, and radiative techniques [12]. However, due to the ineffectiveness of these methods, new approaches have emerged, like targeted cancer therapy [13] with variants that use hyperthermia [14], gene [15] and cancerostatic drug [6] delivery. In particular, the hyperemia generated by absorption of laser light in a range of so-called second biological windows is particularly desired. This relatively new type of therapy is a great alternative for cancer treatment, as it is highly selective and its effects occur in situ without the risk of killing healthy cells and weakening of the immune system [16,17,18]. This promising modality is realized by photoabsorbers, also called photothermal agents. It employs the near-infrared (NIR) laser to generate heat for the thermal ablation of cancer cells. Compared with the conventional therapeutic modalities, photothermal therapy (PTT) exhibits unique advantages in cancer therapy, including high specificity, minimal invasiveness, and precise spatial-temporal selectivity. The most common photothermal agents include gold nanorods and nanospheres, single-wall carbon nanotubes (SWCNTs), graphene oxide (GO), and many others [19,20,21,22,23,24,25,26]. Moreover, carbon nanomaterials such as carbon nanotubes (CNTs) [27] or GO [28] are considered to possess interesting properties for theranostic applications [29,30]. As a matter of fact, carbon nanotubes were used in the PTT of glioblastomas, with good outcomes [20]. However, the biocompatibility of carbon nanotubes has been called into question many times [31,32]. Discovered in the 1960s, nanodiamonds (NDs) are a promising group of carbon nanomaterials which can be successfully utilized in biomedical applications [33]. They are nanometer-sized carbon particles that, due to their inherent properties, have been attracting the interest of researchers in recent years [34,35]. Nanodiamonds exhibit good biocompatibility and low genotoxicity and cytotoxicity in comparison to the dubious compatibility of CNTs [36,37,38]. It was also demonstrated that they do not display any toxic effect to brain cells when they were used for their imaging [39,40]. Furthermore, the functional groups present on the surface of NDs allow for facile conjugation of biological moieties such as proteins [41], DNA [7], RNA [42], or drugs [43]. Lately, the PTT properties of NDs have been recognized as an interesting group of carbon-based photoabsorbers [18]. Therefore, they can pose a great opportunity to develop a whole new family of nanoparticles (NPs) for cancer treatment.

Polydopamine (PDA) is a well-known, mussel-inspired biopolymer that is obtained by the oxidative polymerization process of dopamine in alkaline conditions. It can be deposited on almost any kind of surface [10,44,45]. Apart from its coating abilities and high biocompatibility, PDA increases the water dispersibility of covered particles [46,47,48]. Moreover, PDA exhibits a broad absorption spectrum, especially in the first biological window [36,49,50]. Therefore, PDA can be successfully utilized as an efficient and versatile photothermal agent in PTT therapy [10,49,51]. Through PDA-coating of the base material, PDA allows to increase the PTT properties. Additionally to this, we have recently shown that PDA also provides imaging capabilities, due to the electron scavenging and paramagnetic centers generated in the polymer [52], providing extra functionality.

In this work, we describe a straightforward, facile two-step synthesis of NDs rimmed with PDA and their application in PTT therapy of glioblastoma multiforme. We also demonstrate that PDA coating significantly enhances the PTT properties of different batches of NDs. Moreover, the materials’ properties can be improved by the deposition of indocyanine green (ICG), which is a known fluorescent/thermographic agent [53,54]. The presented data are of great importance for the field of multifunctional materials based on PDA and shed new light on the application of NDs in nanomedicine, with a particular focus on PTT therapy.

## 2. Materials and Methods

### 2.1. Preparation of NDs@PDA

The carboxylated NDs (uDiamond^®^ Vox P) were purchased from Carbodeon (Vantaa, Finland). Hydrogenated and nitrogenized NDs of laser synthesis (NDs-AL), as well as alkylated and siliconized NDs (NDs-BM) were purchased from Ray Techniques Ltd. (Jerusalem, Israel). These were used in order to compare photothermal properties of NDs of different source and type. Nanodiamonds (50 mg) were added into 5 mL of dimethyl sulfoxide (DMSO) and sonicated for 15 min in a horn sonicator (Branson Digital Sonifier SFX 550, Carouge, Switzerland) at 150 W. Nanodiamonds in the DMSO mixture were topped with 95 mL of tris(hydroxymethyl)aminomethane (TRIS) buffer (pH 8.0). Dopamine hydrochloride (50 mg) (Alfa Aesar, Gdansk, Poland) was added to the NDs solution and stirred at 1000 rpm for 24 h at room temperature. The resulting NDs@PDA solution was centrifuged (5810R centrifuge, Eppendorf, Hamburg, Germany) and washed with Milli-Q water three times in order to get rid of the excess unconjugated PDA. 

### 2.2. Indocyanine Green Conjugation 

NDs@PDA aqueous solution at a concentration of 1 mg/mL was combined with ICG solution at 2 mg/mL (the ratio was kept at 1:4, with the total volume being 10 mL). The pH value was set to 2.5, and the mixture was stirred (400 rpm) at room temperature for 24 h in darkness. The resulting dark-green solution was centrifuged for 30 min at 3000× *g* (5810R centrifuge, Eppendorf) and washed three times with Milli-Q water. Remaining particles were dispersed in Milli-Q water once more and kept in darkness.

### 2.3. Spectroscopy Measurements

Fourier-transform infrared spectroscopy (FTIR) of NDs was performed on a Bruker Tensor II (Bruker, Ettlingen, Germany). Pristine NDs, PDA, and NDs@PDA were allowed to desiccate, briefly grinded with KBr and then pressed into a pellet. The signal from a pure KBr pellet was subtracted as background. Ultraviolet–visible–near-infrared (UV–Vis–NIR) spectra were recorded on a Perkin Elmer Lambda 950 UV–Vis Spectrophotometer (Waltham, MA, USA) in the range of 200–1000 nm.

### 2.4. Size Determination of NDs-Based Materials and Zeta Potential

The size of NDs and NDs@PDA conjugates was determined by means of dynamic light scattering (DLS; Litesizer™ 500, Anton Paar, Graz, Austria) and confirmed with high-resolution transmission electron microscopy (HRTEM; JEM-ARM200F, JEOL, Tokyo, Japan) and atomic force microscopy (AFM) in ScanAsyst mode (Bruker, AFM Icon, Hamburg, Germany). The ImageJ software [55] was used to process HRTEM and AFM micrographs in order to analyze the size of NDs and NDs@PDA conjugates. Additionally, the zeta potential of preprocessed NDs was determined in a particle analyzer (Litesizer™ 500, Anton Paar).

### 2.5. Photothermal Measurements

Up to four different concentrations of NDs, NDs@PDA, and NDs@PDA@ICG (25, 50, 100, 200 µg/mL) were irradiated with an 808 nm laser (2 W, Changchun New Industries Optoelectronics Tech. Co., Ltd., Changchun, China). Temperature measurements were collected with a 10 s interval over a 5 min period.

### 2.6. ICG Release

A solution of NDs@PDA@ICG was centrifuged in order to obtain 2 mg of the material. The supernatant was discarded and replaced with Dulbecco’s modified Eagle’s medium (DMEM, Sigma-Aldrich, Sigma-Aldrich, Poznan, Poland). The solution was kept at 37 °C for a period of 24 h in a thermo-shaker (Grant Instruments Ltd., Cambridgeshire, UK). After 2 and 24 h, the sample was centrifuged and the supernatant was collected and replaced with a fresh batch of DMEM. Ultraviolet–visible–near-infrared spectra of the liquid phase were collected in order to assess the ICG release.

### 2.7. Cytotoxicity of NDs-Based Clusters

The glioblastoma multiforme-derived human U-118 MG (malignant glioma) cell line from the American Type Culture Collection (ATCC, Manassas, VA, USA) and human fibroblast cell line MSU1.1 from Prof. Claudine Kieda (Centre de Biophysique Moléculaire (CBM), Centre National de la Recherche Scientifique (CNRS), Orleans, France) were cultured in DMEM (Sigma-Aldrich) supplemented with 10% fetal bovine serum (FBS; Sigma-Aldrich) and 1% antibiotics (penicillin 100 U/mL, streptomycin 100 µg/mL; Sigma-Aldrich) under standard conditions (37 °C, 5% CO_2_). A WST-1 cell proliferation assay (Clontech, Fremont, CA, USA) was carried out to assess the cytotoxicity of bare NDs, NDs@PDA, and NDs@PDA@ICG. The WST-1 cell proliferation assay is based on the enzymatic cleavage of the tetrazolium salt WST-1 to a water-soluble formazan dye, which can be quantified by absorbance at 420–480 nm by live cells. U-118 MG and MSU1.1 cells were seeded at a density of 8 × 10^3^ and 1 × 10^4^ cells per well in a 96-well plate, respectively. After 24 h, the increasing concentrations of all tested nanoparticles (1.25, 2.5, 5, 10, 20, 40 µg/mL) were added to each well, and the cells were incubated for 24 h. Then, 10 µL of the WST-1 cell proliferation reagent was added to each well and incubated for 4 h. After this time, 100 µL of the medium was transferred to new wells and the absorbance was recorded at 450 nm (reference wavelength 620 nm) against the background control, using a multiwell-plate reader (Zenyth, Biochrom, Cambridge, UK). The cell viability was expressed as the respiration activity normalized to untreated cells. All experiments were carried out in triplicate. 

### 2.8. Glioblastoma Multiforme Cells Irradiation

In order to assess the effectiveness of the obtained materials in photothermal therapy after NIR irradiation, U-118 MG cells seeded at a density of 8 × 10^3^ cells per well in 96-well plates were incubated with an increasing (1.25, 2.5, 5, 10, 20, 40 µg/mL) concentration of NDs, NDs@PDA, and NDs@PDA@ICG. After 4 h of incubation, the cells were irradiated by an 808 nm laser with power densities at 2 W for 5 min. The cells were further incubated for 24 h and two cell viability tests were performed: the WST-1 cell viability assay and flow cytometric analysis using the MUSE^®^ count and viability kit (Merck Millipore, Burlington, MA, USA). In the MUSE^®^ count and viability assay, both viable and nonviable cells were differentially stained based on their permeability to the DNA-binding dyes in the reagent. Briefly, cells were trypsinized and suspended in phosphate-buffered saline (PBS). Next, 20 µL of cells in suspension was mixed with 180 µL of the MUSE^®^ count and viability reagent, followed by 5 min incubation at room temperature in the dark. The stained cells were analyzed using the MUSE^®^ cell analyzer (Merck Millipore). Nonirradiated cells incubated with NDs, NDs@PDA, and NDs@PDA@ICG were used for comparison.

To further visualize live and dead cells, U-118 MG cells seeded at a density of 4.5 × 10^4^ cells per well in a 24-well plate were incubated with 40 µg/mL of NDs@PDA@ICG. After 4 h of incubation, the cells were irradiated by an 808 nm laser with a power of 2 W for 5 min. After another 24 h LIVE/DEAD assay was performed. The cells were labeled with 2 µM calcein AM and 2 µM ethidium homodimer-1 containing 250 µL/well of Dulbecco’s phosphate-buffered saline (DPBS; Sigma-Aldrich) for 30 min at 37 °C. Finally, the cells were visualized using the IN Cell Analyzer 2000 (GE Healthcare Life Sciences, Pittsburgh, PA, USA).

### 2.9. Statistical Analysis

Statistical analyses of cell viability results were performed using STATSOFT Statistica 10 software (StatSoft Power Solutions, Inc., Tulsa, OK, USA) and factorial analysis of variance (ANOVA) with a post-hoc Fisher least significant difference (LSD) test. Statistical significance was assumed for *p*-value < 0.05.

## 3. Results

### 3.1. Characterization of NDs, NDs@PDA, and NDs@PDA@ICG

**Figure 1:**
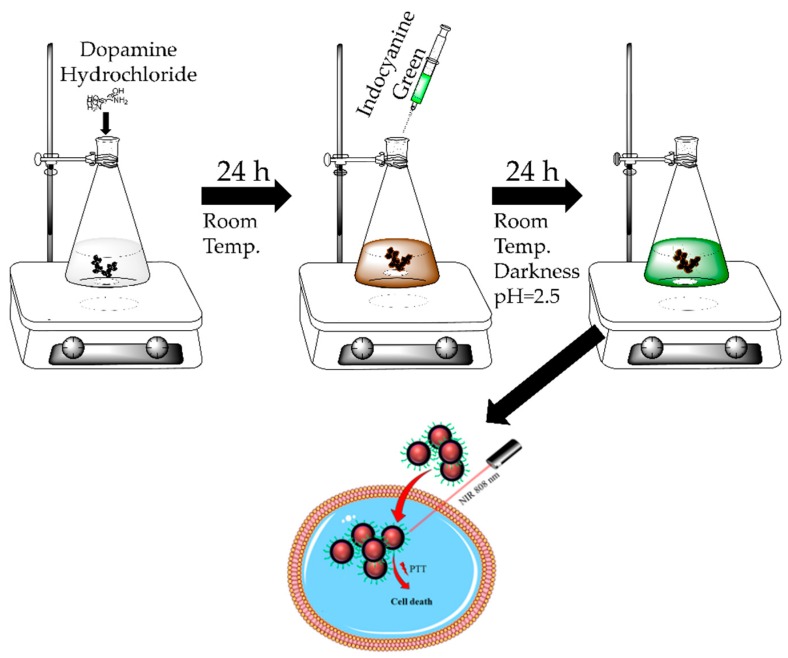
Schematic of the synthesis of polydopamine (PDA)-coated nanodiamonds (NDs) with indocyanine green (ICG) conjugation, and its application in the photothermal therapy (PTT) of the glioblastoma cell.

Initially, NDs were briefly sonicated in 5 mL of DMSO in order to obtain a good dispersion and lessen the size of the NDs clusters. After sonication, NDs were coated with a PDA layer under basic conditions using a dopamine solution of 2 mg/mL. Starting turbid and slightly milky, the solution of NDs in TRIS buffer turned to yellow, then brown, and finally into black while polymerization was occurring. Obtained NDs@PDA conjugates were then mixed with aqueous solution of ICG and kept stirring in darkness for 24 h in an acidic solution (pH 2.5). The entire process is shown schematically in Figure 1. The nanomaterials were investigated by a variety of techniques to verify the coating of PDA on top of the NDs, to determine the morphology of NDs and NDs@PDA, as well as to ascertain ICG conjugation.

**Figure 2:**
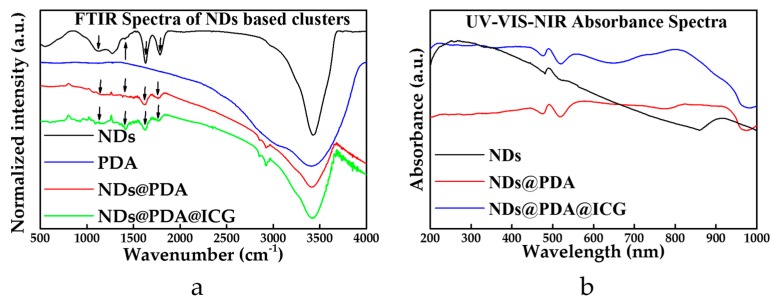
(**a**) FTIR spectra of NDs (black), PDA (blue), NDs@PDA (red), and NDs@PDA@ICG (green). Arrows indicate the characteristic bands in PDA and NDs, allowing assessment of a successful functionalization process. (**b**) UV–Vis–NIR absorbance spectra of NDs (black), NDs@PDA (red), and NDs@PDA@ICG (blue). The NDs@PDA@ICG exhibits the highest absorption for NIR irradiation (797 nm). a.u.: Arbitrary units.

**Figure 3:**
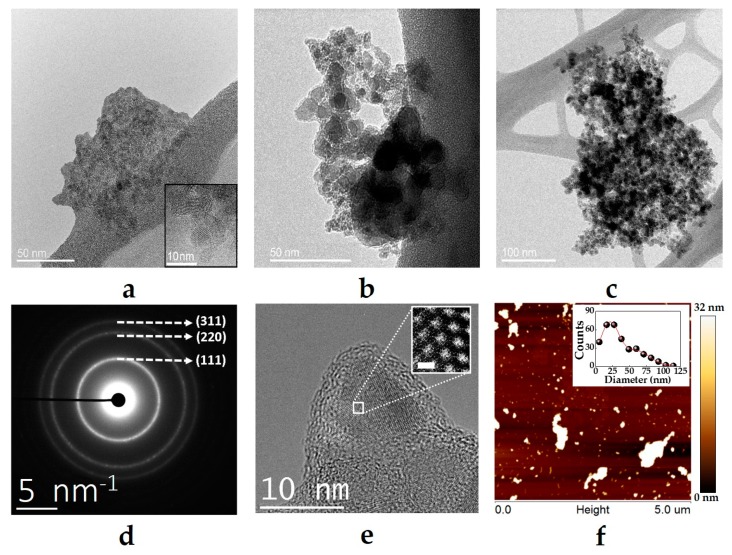
HRTEM images of (**a**) pristine NDs, (**b**) NDs@PDA, and (**c**) NDs@PDA@ICG. Inset in (**a**) shows the crystalline character of NDs with the graphitic surface. In the NDs@PDA and NDs@PDA@ICG, we could see the PDA layer encompassing the carbon nanoparticles. (**d**) The selected area diffraction pattern from the pristine nanoparticles. (**e**) HRTEM image of a single NDs, where the inset shows a high magnification of the NDs (scale bar: 1.54 Å). (**f**) AFM micrograph of NDs on mica sheet. The inset shows the size distribution of NDs clusters. The biggest clusters were excluded from the diameter measurements because of their irregular shape, as the diameters were obtained from ImageJ cell count function.

The successful formation of the PDA layer on NDs was proved by FTIR (Figure 2a). In the recorded spectra of PDA, the absorption band at 3415 cm^−1^ was assigned to the stretching vibrations of –OH and N–H groups in the PDA. However, this band was slightly downshifted in the spectrum of NDs@PDA to 3405 cm^−1^. The peak at 3425 cm^−1^ in the spectrum of pristine NDs was assigned to the same vibrational modes as they were obtained in a detonation method, in which oxygen and nitrogen can be easily incorporated into the structure of NDs. The signals at 1617 cm^−1^ and 1630 cm^−1^ of pristine NDs and PDA, respectively, belonged to the C=C groups from carbon moieties present in bare NDs and bending vibrations of amines from PDA. This peak remained almost unaffected in the PDA-coated NDs and appeared at 1627 cm^−1^. However, its origin in the composite spectra was hard to assign due to the overleaping bands from different groups present in this region. The peak at 1784 cm^−1^ corresponded to a stretching vibration of C=O moiety present in the spectrum of mere NDs, which was strongly attenuated in the final composites with the PDA layer (Figure 2a). The signals from the C=N and C=C stretches at 1515 cm^−1^ in NDs@PDA were less intense compared to pure PDA due to the signal drop for NDs in that area. The C–O signal at 1292 cm^−1^ for PDA and 1274 cm^−1^ for NDs was slightly upshifted in the conjugate (1299 cm^−1^). The C=N band at 1400 cm^−1^ for PDA was also upshifted to 1404 cm^−1^ (Figure 2a, green arrows). More interestingly, we observed a slight bulge at that wavenumber for NDs, which might indicate some nitrogen incorporation in the diamond structure which correlates to its fluorescent abilities [56]. The 1130 cm^−1^ O–H bending present in the NDs was also apparent in the NDs@PDA composite (Figure 2a, black arrows). In the case of the NDs@PDA@ICG, no obvious differences between the final product and NDs@PDA in the FTIR spectra (Figure 3a) were observed apart from a slight attenuation of the signal.

In the next step, absorbance spectra of the nanomaterials were collected in order to assess their absorption efficiency in the UV–Vis–NIR region (Figure 2b). The high absorbance of UV light (Figure 2b, black line) for NDs originated from a relatively high bandgap of around 3.5 eV [57]. The absorption in the NIR region slightly increased for NDs@PDA (Figure 2b, red line), but the biggest absorbance readout occurred at 797 nm with the presence of ICG in the final product (Figure 2b, blue line). This result proved the successful conjugation of ICG to the NDs@PDA surface. The interaction between the PDA shell and ICG occurred probably due to the π–π interaction of aromatic rings present in both structures. Moreover, the hydrogen bonding could have been involved in this interaction too [58].

Equipped with data from FTIR, we evaluated the colloidal behavior of pristine NDs, NDs@PDA, and NDs@PDA@ICG samples to see the influence of PDA and ICG on the stability of NDs. The zeta potential determined for pristine NDs in water was −38 mV; thus, they exhibited high colloidal stability, which is in agreement with the Derjaguin−Laudau−Verwey−Overbeek (DLVO) theory which states that the colloidal stability of the system is maintained as the zeta potential is below −30 mV [59,60]. After coating with PDA, the zeta potential went up to −28 mV. This value is in agreement with the zeta potential for other PDA-coated nanomaterials already reported in the literature, which additionally confirmed the rimming of NDs with PDA. However, the NDs@PDA composite has a slightly higher tendency to precipitate in comparison to pristine NDs, since the zeta potential was raised slightly above −30 mV (Table 1). However, dispersion of NDs@PDA remained stable for a couple of hours and could simply be redispersed by handshaking. According to other reports concerning ICG conjugation [61], the zeta potential should stay unchanged or changes insignificantly; thus, we did not perform appropriate measurements. Dynamic light scattering measurements showed that NDs aggregate, creating clusters which appeared to be 255.47 ± 8.19 nm in diameter. The coating of NDs with PDA influenced the hydrodynamic diameter, since the size diminished to 228.27 ± 3.76 nm along with the decrease in polydispersity index, from 0.36 ± 0.01 to 0.24 ± 0.01. After ICG conjugation, the size increased to 357.57 ± 8.63 nm; however, the polydispersity index remained almost unchanged, at 0.25 ± 0.02.

The HRTEM was used to ascertain the size of all the samples. The pristine NDs samples consisted of clusters with various sizes, ranging from a few dozens of nanometers up to a few microns in diameter (Supplementary Figure S1a). Despite the sonication, the diameters of the aggregates did not decrease significantly (Figure 3a). However, this is an effect which commonly occurs during sample preparation. The size obtained with DLS (See Table 1) for NDs and their derivatives was generally below 400 nm. High-resolution transmission electron microscopy imaging proved that NDs were coated collectively with PDA, which led to a certain degree of increase in diameter (Figure 3b and Supplementary Figure S1b). Before PDA and ICG conjugation, the solutions were sonicated for only 15 min. This method, however, did not yield single-digit NDs. The samples were inhomogeneous in terms of diameters. The addition of PDA, and a decrease in the size of NDs@PDA conjugates in comparison to pristine NDs (Figure 3a,b) can be related to the high stability of PDA in aqueous solutions. The addition of ICG, on the other hand, causes a bigger than expected diameter increase of the final product (Table 1, Figure 3c, and Supplementary Figure S1c).

The size of the NDs clusters was additionally confirmed with AFM measurements (Supplementary Figure S2). A single drop of NDs solution was cast on a mica sheet and dried for a few hours before the measurement. As shown in Figure 3f, the NDs appeared both as clusters and presumably single-digit NDs. The size of the clusters varies from a few nanometers up to over 1 µm. It is likely that this is caused by the desiccation of the sample during preparation for observations. As the water’s volume decreases distance between NDs diminishes and mutual attraction becomes stronger hence the aggregates grew in size. The size distribution presented in Figure 3f shows a bimodal character with diameters between 21 and 60 nm. The micron-scale clusters were not presented on the graph due to their irregular shapes, which give an overestimated size in the evaluation process using the ImageJ software.

### 3.2. Photothermal Properties

**Figure 4:**
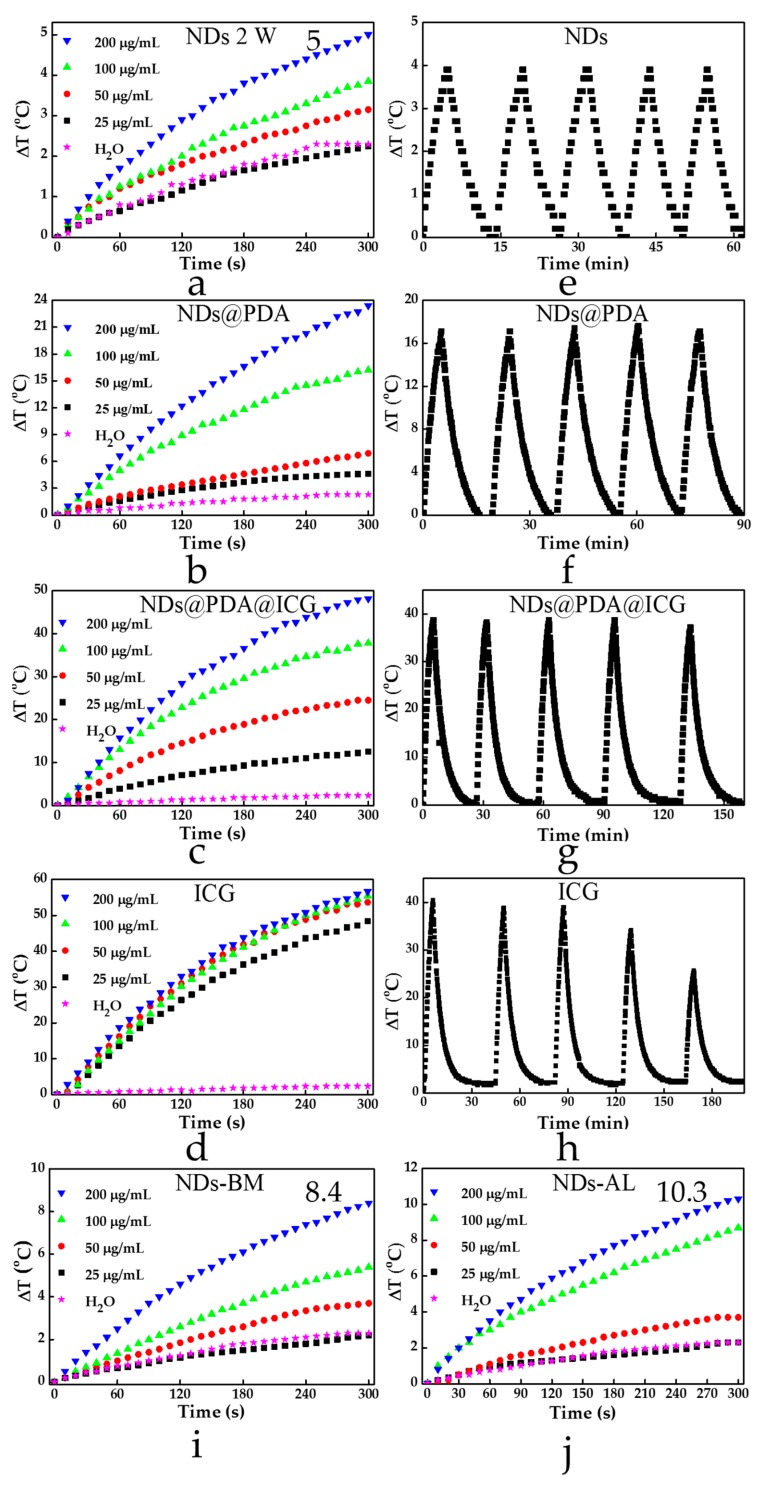
(**a**–**d**) Increase in solutions’ temperature in time after NIR laser irradiation for different concentrations of (**a**) NDs, (**b**) NDs@PDA, (**c**) NDs@PDA@ICG, and (**d**) ICG alone. (**e**–**h**) Stability of (**e**) NDs, (**f**) NDs@PDA, (**g**) NDs@PDA@ICG, and (**h**) ICG at 100 μg/mL after five cycles of irradiation. (**i**,**j**) The photothermal response of (**i**) NDs-BM (alkylated and siliconized; detonation NDs) and (**j**) NDs-AL (hydrogenized and nitrogenized; laser NDs). They exhibit slightly better photothermal properties in comparison to carboxylated NDs. Maximal temperature increase is indicated for (**a**) NDs, (**i**) NDs-BM, and (**j**) NDs-AL at a concentration of 200 µg/mL reaching 5, 8.4, and 10.3 °C, respectively.

**Figure 5:**
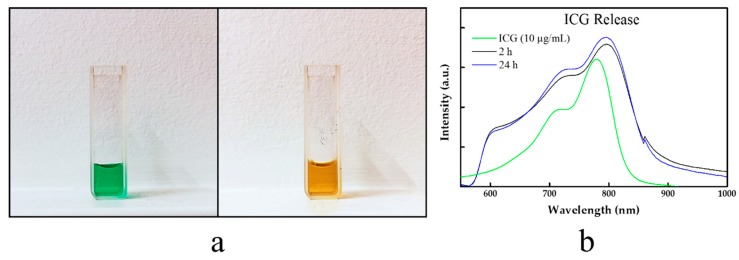
(**a**) The color change of ICG solution after NIR irradiation. Fresh ICG solution (left) and ICG solution after five irradiation cycles (right). (**b**) UV–Vis–-NIR spectra of supernatant collected after 2 and 24 h incubation of NDs@PDA@ICG at 37 °C in cell culture medium. Each spectrum corresponds to bare ICG (10 µg/mL; green), ICG released from NDs@PDA@ICG to the medium after 2 h (black) and 24 h (blue). As the intensity of the signal does not differ significantly between the measurements at 2 and 24 h, it is likely that ICG is stably attached to the NDs@PDA surface. a.u.: Arbitrary units.

It is worth highlighting that in a recent paper, Ryu et al. [18] reported strong photothermal properties of detonation NDs with a carboxylated surface; those NDs under irradiation with an 808 nm laser (2 W) could increase the temperature by around 18 °C after 5 min of irradiation at a concentration as low as 1 µg/mL. Therefore, we investigated the absorption of NIR light of pristine NDs to see whether they could be considered as efficient photothermal agents. In the performed experiments, a pristine NDs sample was irradiated with a laser beam of 808 nm at a power of 2 W for 300 s. However, the commercial NDs employed for this test showed a very weak photothermal response, and we could not observe this phenomenon at such a low NDs concentration as reported in [18]. The first small temperature increase was observed when the dispersion of NDs at a concentration of 100 μg/mL was submitted to laser irradiation, resulting in a temperature increase by ca. 4 °C (Figure 4a). Under the same conditions, the water temperature increased by 2 °C. There was a further NDs increase to 200 μg/mL, resulting in a higher photothermal response since the average temperature was increased by about 5 °C. Polydopamine is known to possess strong absorption in the NIR region; thus, it has lately been used as a photothermal agent in cancer therapy. Therefore, we expected that NDs@PDA would assure stronger absorption of NIR light and better photothermal properties at a lower dosage in comparison to pristine NDs. Indeed, the obtained NDs@PDA nanomaterial exhibited photothermal properties even at a low concentration of 25 µg/mL, since the medium temperature was increased by about 3 °C under NIR irradiation conditions (Figure 4b). Further increase in concentration to 50 μg/mL and 100 μg/mL resulted in raising the temperature by 6 °C and 16 °C, respectively. At a concentration of 200 μg/mL, the medium temperature was elevated by almost 24 °C under NIR irradiation in contrast to pristine NDs, which showed only an increase by 5 °C. This happened because with an increased mass of composite we increased the amount of PDA, which is mostly responsible for photothermal properties. The conjugation of ICG in the final product elevated the temperature even higher, resulting in an almost 50 °C temperature increase for a 200 µg/mL sample. Even for the lowest concentration of 25 µg/mL, the difference was around 10 °C (Figure 4c). Considering the fact that only ca. 25% of the initial ICG was conjugated (ICG uptake calibration curve estimated loading), we were able to obtain very good results for the NDs@PDA@ICG. On the other hand, since human body temperature is around 36.6 °C, the resulting increase was enough to induce hyperthermia, resulting in cancer cell death. Furthermore, the photostability test of NDs (Figure 4e), NDs@PDA (Figure 4f), NDs@PDA@ICG (Figure 4g), and ICG alone (Figure 4h) was checked. The performed tests revealed that even though free ICG caused the highest temperature change, it suffered from photobleaching. Thus, its repeated usage is questionable, since we observed that after the third cycle, the temperature increase was not as high as in previous runs (Figure 4g). Moreover, the solution color changed from green to orange-brown, due to the degradation of ICG under NIR irradiation (Figure 5). It is worth highlighting that both NDs@PDA and NDs@PDA@ICG could be used in at least five on/off cycles of laser irradiation, which proved their high photothermal stability and improved photostability of ICG after linkage to PDA-coated NDs.

Since the photothermal response of carboxylic, detonation NDs (5 °C at 200 µg/mL) was much lower than expected, we decided to compare these with two other types of pristine, commercially available NDs. However, none of the materials exhibited such a high photothermal growth as could be found in the literature. Both types of NDs provided by Ray Techniques Ltd. exhibited a slightly better photothermal response (8.4 °C for NDs-BM and 10.3 °C for NDs-AL at 200 µg/mL) in comparison to investigated NDs. Moreover, even the temperature of 10.3 °C for NDs-AL that was obtained only for a high concentration of 200 µg/mL, which is a concentration 200 times higher than previously reported and still yielded a photothermal response that was almost two times lower [18].

In our study, we also checked the release of ICG from the obtained materials. The NDs@PDA@ICG was incubated in cellular medium at 37 °C, and then the supernatant was collected after 2 and 24 h to investigate its release from the carrier. The performed studies revealed indeed that the ICG at a given condition was released from the nanocarrier, which is also a known phenomenon for other PDA-based nanostructures. However, those reports revealed that the presence of PDA hampers the ICG release from the carrier, which is the desired effect [62,63].

Finally, based on the equation reported by Liu et al. [64], we assessed the photothermal conversion efficiency (*η*) of our materials as follows:

(1) $$\eta =\frac{hA\Delta T_{\max}-Q_{s}}{I\left(1-10^{-A}\lambda \right)},$$

where *h* is the heat transfer coefficient, *A* is the surface area of the container, Δ*T*_max_ is the temperature change of the NPs solution at the maximum steady-state temperature, *I* is the laser power, *A_λ_* is the absorbance of different NPs at 808 nm, and *Q_s_* is the heat associated with the light absorbance of the solvent. 

Nanodiamonds themselves showed a low photothermal conversion efficiency at a level of 8.7%, but the addition of PDA (36.5%) and ICG (44.5%) raised the efficiency close to and slightly above the level of 40% reported by Liu et al. [64]. The low conversion efficiency of NDs-BM (5.4%) and NDs-AL (5.7%) is closely related to their absorbance at 808 nm, which, in return, is the outcome of enhanced scattering in the solution (0.9 and 1.3, respectively, compared to 0.3 of pristine NDs). All heat transfer coefficients of different materials are presented in Table 2.

### 3.3. Cell Viability and In Vitro PTT of Glioblastoma Cells

**Figure 6:**
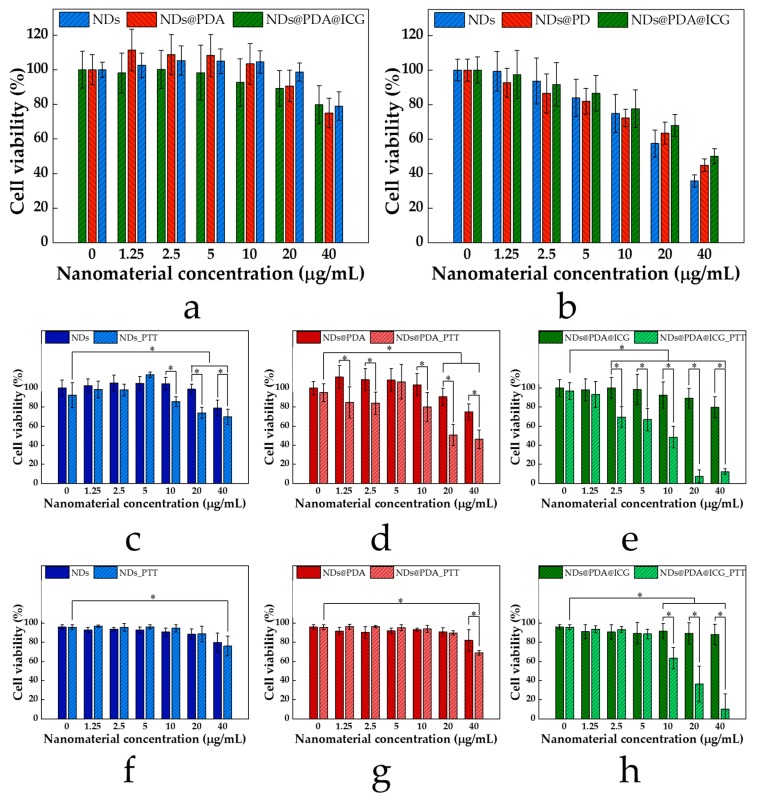
WST-1 viability results of NDs-based clusters in (**a**) U-118 MG and (**b**) MSU 1.1 cells. (**c**–**e**) WST-1 cell viability with and without laser irradiation. (**f**–**h**) Count and viability assay results of NDs-based clusters with and without laser irradiation. Error bars represent the standard deviation. *, *p*-value < 0.05.

The cytotoxicity of pristine NDs, NDs@PDA, and NDs@PDA@ICG were evaluated in vitro using a WST-1 cell proliferation assay. The results are shown in Figure 6. For the cytotoxicity tests, NDs, NDs@PDA, and NDs@PDA@ICG in concentrations up to 40 µg/mL were cultivated with U-118 MG (Figure 6a) and healthy MSU1.1 (Figure 6b) cells for 24 h in standard conditions at 37 °C. 

The glioblastoma cells showed concentration-dependent mortality when cultivated with all aforementioned types of materials (Figure 6a) since the cell viability decreased from around 100% at 1.25 µg/mL for each sample to slightly below 80% (78.99% for NDs, 74.97% for NDs@PDA, and 79.76% for NDs@PDA@ICG) at a concentration of 40 µg/mL. For fibroblast cells, the concentration-dependent toxicity was also observed for each type of NDs (Figure 6b).

The activity of NDs, NDs@PDA, and NDs@PDA@ICG in the photothermal therapy of glioblastoma cells was determined using WST-1 (Figure 6c,d) and flow cytometry analysis (Figure 6e–h and Supplementary Figure S3). Even though pristine NDs showed weak photothermal properties, we assessed their usage in PTT therapy of glioblastoma. As expected, NDs caused a feeblish effect on cells’ viability in the applied concentration range under NIR irradiation conditions. After irradiation with a laser beam at the highest concertation, 40 µg/mL of NDs’ cell viability dropped around 20%. These data are consistent with the results from the photothermal test, which indicated a very weak NIR response of these commercially available NDs (See Figure 4).

Furthermore, we studied the cytotoxicity of NDs@PDA composites using the same concentration range. Interestingly, in contrast to pristine NDs, the NDs@PDA composite showed a slightly higher toxic influence on glioblastoma cells for concentrations above 10 µg/mL. However, the viability pertained high levels even for the concentration of 40 µg/mL, where the cell viability went down by only 20% (75% WST-1, 81% count and viability assay). This result is in agreement with recently published studies, which have demonstrated that PDA-coated magnetic nanostructures do not exhibit a cytotoxic effect on cancer cells [65].

Since NDs@PDA showed stronger photothermal properties than pristine NDs, we investigated the efficacy of PTT on glioblastoma cells as well. The cancer cells were incubated with NDs@PDA for 4 h and then irradiated with a laser beam of 2 W for 5 min (Figure 6d,g). 

As shown in Figure 6d,g, the laser irradiation therapy with NDs@PDA was more efficient than in the case of pristine NDs, since the cells’ viability dropped to even around 50%. Apparently, a much stronger PTT effect was observed for concentrations of 20 and 40 µg/mL. At those concentrations, the cell’s viability decreased to around 51% and 46% (89% and 69%; count and viability assay), respectively, demonstrating that higher concentrations were necessary to generate a hyperthermic effect. However, one needs to take into consideration that the effects from pristine NDs at the same concentration were visibly lower. Therefore, PDA-coated NDs were superior to pristine NDs in the performed PTT therapy of glioblastoma.

Lastly, we investigated the influence of irradiation on cells incubated with NDs@PDA@ICG. Since these showed the highest photothermal response with reasonably low cytotoxicity, we expected the best results in terms of photothermally-induced cell death. As in previous cases, the general viability of cells after composite incorporation decreased to only about 80% for the highest concentration of 40 µg/mL (Figure 6e,h). After the irradiation process, the viability decreased to 70% already at a concentration of 2.5 µg/mL and continued to drop, reaching even 7% and 12% for 20 and 40 µg/mL (36% and 10%; count and viability assay), respectively. This unprecedentedly shows that NDs@PDA@ICG composites can serve as effective PTT agents in cancer treatment. We also found that for the highest concentrations (10, 20, 40 µg/mL), there was a statistically relevant difference between the non-irradiated and irradiated samples’ viability.

**Figure 7:**
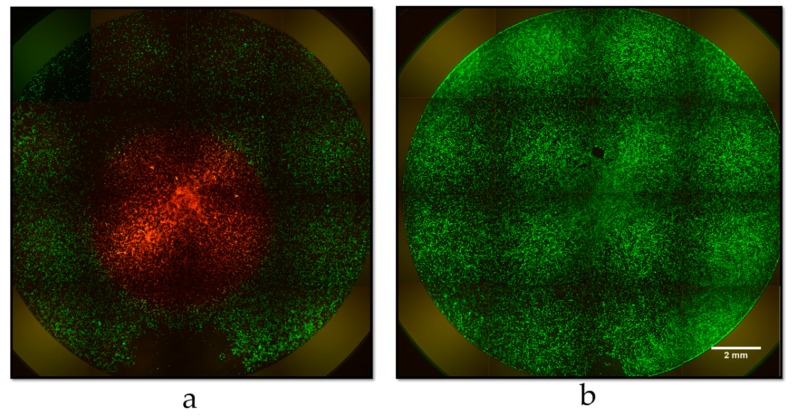
Fluorescence microscopy images of U-118 MG cells incubated with NDs@PDA@ICG (**a**) with and (**b**) without NIR laser irradiation by LIVE/DEAD assay. Alive cells are shown in green, whereas dead cells are shown in red.

Moreover, we investigated the effect of irradiation on cancer cells with a laser beam using a LIVE/DEAD assay. We found out that cells directly irradiated with an NIR beam suffered from increased cytotoxicity compared to those outside the beam (Figure 7a,b); hence, with localized irradiation, cancer will only be killed during the PTT process.

## 4. Discussion

In accordance with previous reports, NDs are generally seen as biocompatible, noncytotoxic, and nongenotoxic [38]. Our results confirm those reports to some extent; however, above a certain concentration (40 µg/mL), their cytotoxicity rises significantly (c.a. 20% WST-1; c.a. 65% count and viability assay). Their photothermal response to different kinds of commercially available NDs is rather low, as the difference between the viability of nonirradiated and laser-treated cells is around 10% (up to c.a. 25% for 20 µg/mL WST-1; 13% count and viability assay). This stands in opposition to previous reports of good photothermal properties of carboxylated NDs [18]. NDs@PDA generally show similar levels of cytotoxicity as pristine NDs [17]. At the same time, NDs@PDA NPs exhibit a better photothermal response [66]. The addition of ICG elevates the photothermal properties (*η* = 44.5%) even further, while maintaining the high biocompatibility of the NPs. Moreover, NDs@PDA@ICG NPs pose the highest viability compared to other ND-based materials in both tests.

Another important factor in utilizing the NPs for in vitro and in vivo applications, such as photothermal therapy, is their colloidal stability. The mean hydrodynamic diameter of the ND agglomerates, 255.47 ± 8.19 nm, slightly diminishes after PDA conjugation to 228.27 ± 3.76 nm, only to rise for NDs@PDA@ICG to 357.57 ± 8.63 nm. The degree of agglomeration can influence the cytotoxicity of the material and is strongly related to NP concentration [67]. This was quite apparent for pristine NDs; however, these agglomerates were most likely created dynamically (especially during desiccation) and can be easily broken down in the sonication process. Even despite the rise in NP diameter in the final product, they still exhibit good biocompatibility; thus, we strongly believe that our system can be utilized as potential PTT agents. Furthermore, investigations of the physical diameter of NDs by HRTEM and AFM show that they are mostly below 200 nm, which allows us to claim that they can be considered in further studies on crossing the blood–brain barrier (BBB). The particles’ size in terms of crossing the BBB is a crucial parameter, as shown in the latest studies on gold nanostructures and their capability to cross the BBB [68,69,70].

Due to the large band gap of NDs, they exhibit a low light-to-heat transformation in the NIR region; thus, they can only be utilized at high concentrations as potential photothermal agents. NDs@PDA and NDs@PDA@ICG, on the other hand, have much better light-to-heat conversion upon NIR irradiation. With the reported fluorescence of ICG [53,54,61], NDs@PDA@ICG can serve as an imaging and therapeutic agent. With good stability after five cycles of irradiation, thermally induced death of cancer cells is still achievable, showing that NDs@PDA@ICG conjugates can be utilized in multiple sessions during therapy.

## 5. Conclusions

Here, we have presented the straightforward preparation of multifunctional nanomaterials for PTT of glioblastoma based on NDs and PDA. The obtained nanostructures were chartered by FTIR, AFM, TEM, UV–Vis–NIR, and DLS, as well as zeta potential. The performed analysis confirmed coating with PDA and showed good stability of synthesized materials. Moreover, the obtained NDs@PDA material exhibited high photothermal conversion photothermal properties that could be further enhanced by deposition of ICG. This poses a tremendous possibility in the planning of PTT sessions with the use of photothermal agents in cancer therapy. Furthermore, both materials had very good photostability, since they could be used in five consecutive cycles of irradiation with a laser beam and did not suffer from photobleaching. Finally, NDs@PDA and NDs@PDA@ICG appeared to be superior photothermal agents in comparison to pristine NDs from different commercial sources, and allowed for the efficient PTT therapy of glioblastoma at low material concentrations, which was investigated by the WST test and flow cytometry. Furthermore, the presence of PDA and ICG in NDs makes them more versatile, since it opens up the possibility to use them in combined photothermal/photodynamic therapy and photoacoustic imaging [71].

## Figures and Tables

**Table 1:** Dynamic light scattering measured the hydrodynamic diameter and zeta potential of NDs-based clusters.

Sample	Size (nm) ^1^	Polydisperisty Index ^1^	Zeta potential (mV)
NDs	255.47 ± 8.19	0.36 ± 0.01	−38.02
NDs@PDA	228.27 ± 3.76	0.24 ± 0.01	−28.06
NDs@PDA@ICG	357.57 ± 8.63	0.25 ± 0.02	-

^1^ The data is presented as the mean ± standard deviation.

**Table 2:** Comparison of photothermal conversion efficiency (*η*) of different NDs-based nanoparticles.

Sample	Δ*T*_max_	*η*
NDs	3.9	8.7%
NDs@PDA	17.1	36.5%
NDs@PDA@ICG	38.7	44.5%
NDs-BM	5.4	5.4%
NDs-AL	8.7	5.7%
ICG	56.6	56.6%

CNSs: Colloidal nanospheres; DPA: Dopamine; ICG: Indocyanine green; NDs: Nanodiamonds; NDs-AL: Hydrogenized and nitrogenized NDs; NDs-BM: Alkylated and siliconized nanodiamonds; PDA: Polydopamine.

## Supplementary Materials

The following are available online at http://www.mdpi.com/2313-7673/4/1/3/s1, Figure S1: HRTEM micrographs of pristine NDs, NDs@PDA, and NDs@PDA@ICG clusters; Figure S2: AFM micrographs of pristine NDs; Figure S3: Representative plots from the MUSE^®^ count and viability assay.
