# Supplementary material for: NDs@PDA@ICG Conjugates for Photothermal Therapy of Glioblastoma Multiforme
Source: Biomimetics (Basel). 2019 Jan 11;4(1):3. doi: 10.3390/biomimetics4010003 (PMC6477600; doi:10.3390/biomimetics4010003)
Supplement: Supplementary file 1 [file biomimetics-04-00003-s001.pdf]

Supplementary Materials: NDs@PDA@ICG Conjugates for Photothermal Therapy of Glioblastoma Multiforme

Damian Maziukiewicz [1,2], Bartosz F. Grześkowiak [1], Emerson Coy [1], Stefan Jurga[1] and Radosław Mrówczyński [1,*]

1 NanoBioMedical Centre, Adam Mickiewicz University, ul. Umultowska 85, PL-61614 Poznań, Poland; damian.maziukiewicz@amu.edu.pl (D.M.); bartoszg@amu.edu.pl (B.F.G.); coyeme@amu.edu.pl (E.C.); stjurga@amu.edu.pl (S.J.)

2 Department of Macromolecular Physics, Faculty of Physics, Adam Mickiewicz University, Umultowska 85, PL-61614 Poznań, Poland

* Correspondence: radoslaw.mrowczynski@amu.edu.pl; Tel.: +48-662-056-999

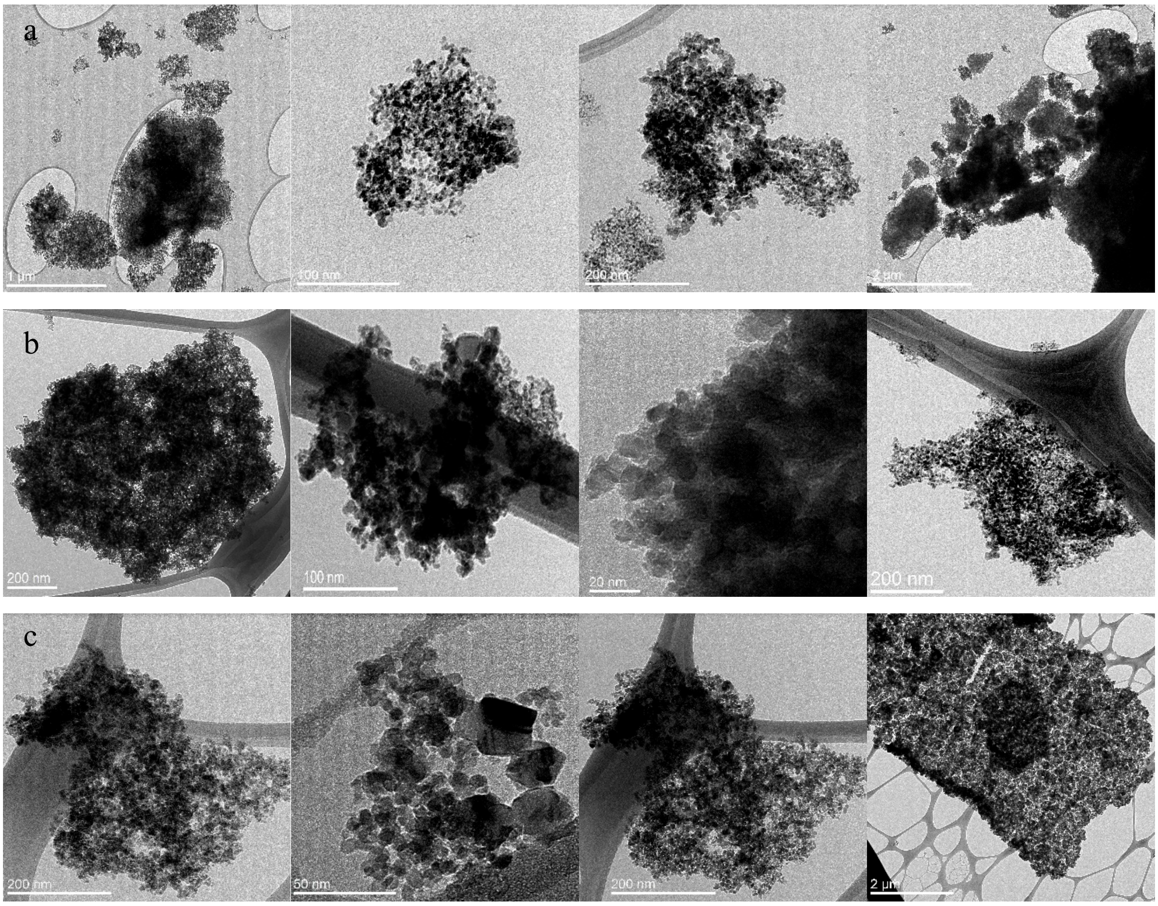

Figure S1. HRTEM micrographs of (**a**) pristine NDs, (**b**) NDs@PDA, and (**c**) NDs@PDA@ICG clusters. Images show the diversity in size of the clusters on every stage of functionalization.

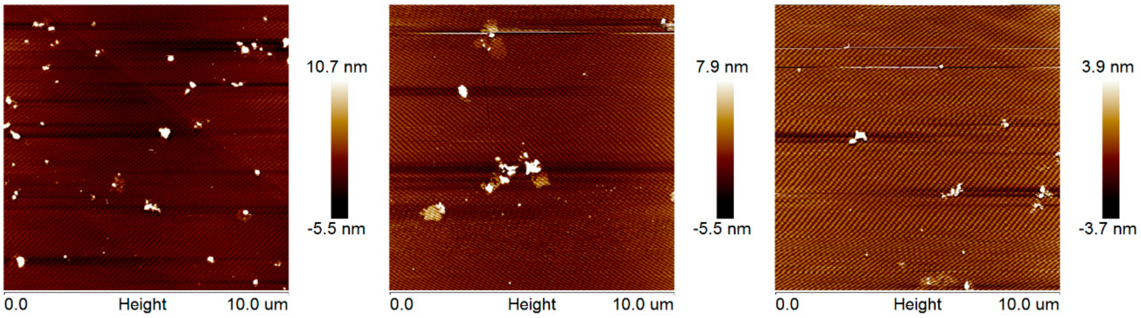

Figure S2. AFM micrographs of pristine NDs.

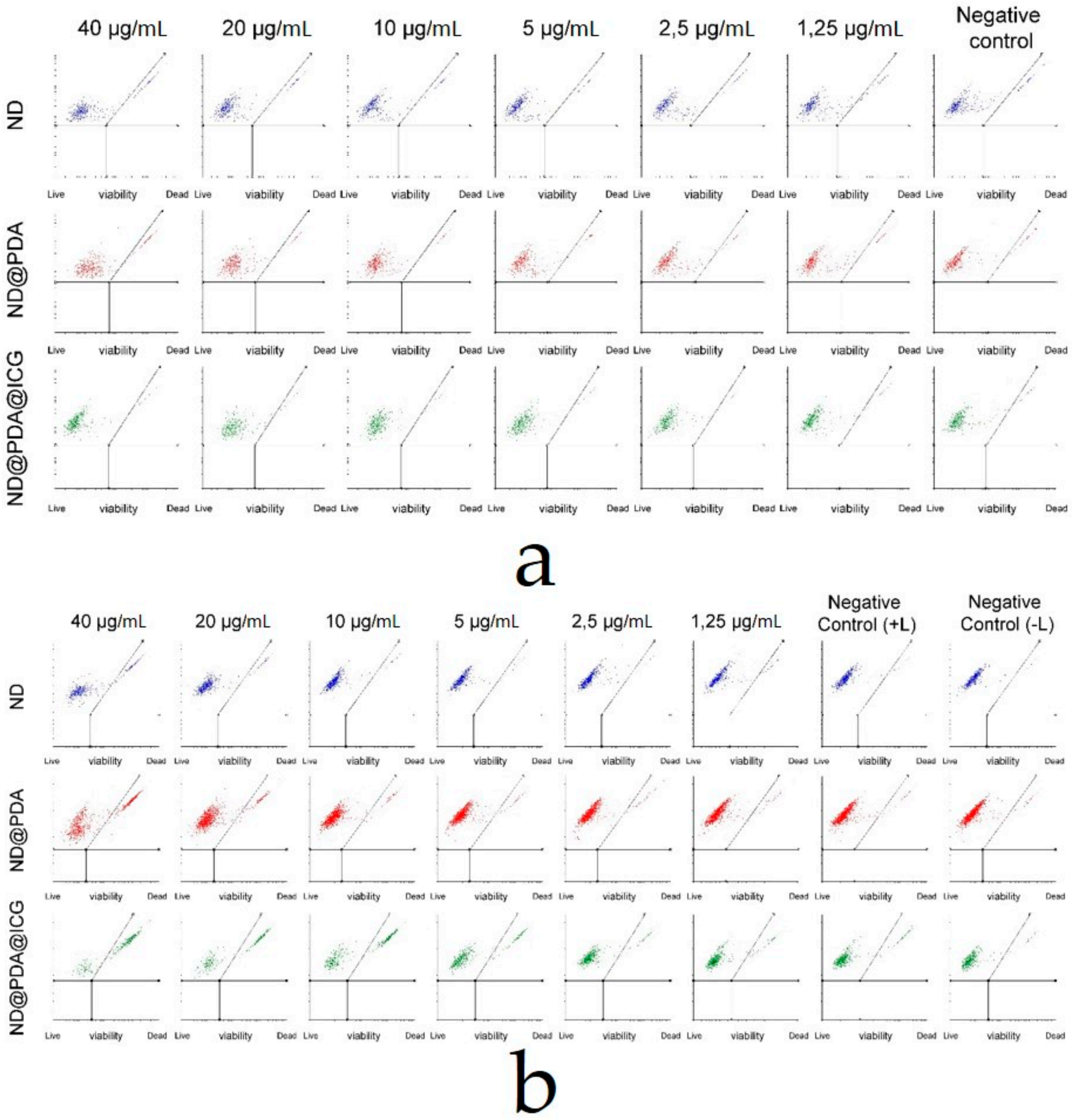

Figure S3. Representative plots from the MUSE® count and viability assay. Results obtained for (**a**) nonirradiated and (**b**) irradiated samples.
